# Mechanisms and pathways to impact in public health research: a preliminary analysis of research funded by the National Institute for Health Research (NIHR)

**DOI:** 10.1186/s12874-020-0905-7

**Published:** 2020-02-19

**Authors:** Harriet Boulding, Adam Kamenetzky, Ioana Ghiga, Becky Ioppolo, Facundo Herrera, Sarah Parks, Catriona Manville, Susan Guthrie, Saba Hinrichs-Krapels

**Affiliations:** 1 The Policy Institute, King’s College London, 22 Kingsway, London, WC2B 6LE UK; 2 RAND Europe, Westbrook Centre, Cambridge, CB4 1YG UK

**Keywords:** Research impact, Public health, Impact pathways, Research impact assessment

## Abstract

**Background:**

The mechanisms and pathways to impacts from public health research in the UK have not been widely studied. Through the lens of one funder (NIHR), our aims are to map the diversity of public health research, in terms of funding mechanisms, disciplinary contributions, and public health impacts, identify examples of impacts, and pathways to impact that existing reporting mechanisms may not otherwise have captured, and provide illustrations of how public health researchers perceive the generation of non-academic impact from their work.

**Methods:**

A total of 1386 projects were identified as ‘public health research’ by the NIHR and listed in the NIHR Public Health Overview database (2000–2016). From these, a subset of 857 projects were matched as potentially having begun reporting impacts via an external data-gathering platform (Researchfish). Data on the 857 projects were analyzed quantitatively, and nine projects were selected to investigate further through semi-structured interviews with principal investigators. Two workshops took place to validate emerging and final findings and facilitate analysis.

**Results:**

In addition to the NIHR School for Public Health Research and the NIHR Public Health Research Programme, 89% of projects contained in the NIHR Public Health Overview portfolio as ‘public health research’ are funded via other NIHR research programmes, suggesting significant diversity in disciplines contributing to public health research and outcomes. The pathways to impact observed in our in-depth case studies include contributing to debates on what constitutes appropriate evidence for national policy change, acknowledging local ‘unintended’ impacts, building trusted relationships with stakeholders across health and non-health sectors and actors, collaborating with local authorities, and using non-academic dissemination channels.

**Conclusions:**

Public health as a discipline contributes substantially to impact beyond academia. To support the diversity of these impacts, we need to recognise localized smaller-scale impacts, and the difference in types of evidence required for community and local authority-based impacts. This will also require building capacity and resources to enable impact to take place from public health research. Finally, support is required for engagement with local authorities and working with non-health sectors that contribute to health outcomes.

## Background

‘Impact’ from academic research can be defined differently by policy and practice organisations, academia, commissioners, and funders, but it broadly refers to any change or benefit to society beyond producing academic publications. For example, UK Research and Innovation (UKRI) refers to impact as “the demonstrable contribution that excellent research makes to society and the economy”.^1^ For the purposes of the UK 2014 and 2021 Research Excellence Framework (REF), impact is defined as “an effect on, change or benefit to the economy, society, culture, public policy or services, health, the environment or quality of life, beyond academia”. Analysis and assessment of these wider impacts from research allows for better allocation of research funding, creates accountability for research, and supports advocacy initiatives in policy and practice [1]. In the case of public health research, few studies have been conducted that specifically demonstrate the contribution that the field has made to society. This may be partly due to the scarcity of research impact analyses generally, or, specifically for public health, due to the diversity of ways in which the field is described. Although there is no global, organization-wide definition of ‘public health’, a common point for scoping is the population health approach and the production of generalizable knowledge for future interventions [2]. For example, one of the most widely used definitions is that of the WHO based on the work of Acheson [3], where public health is defined as “the science and art of promoting and protecting health and wellbeing, preventing ill health and prolonging life through the organised efforts of society”. As such, there are many disciplines that could potentially contribute to what may be considered public health benefits to society, or ‘public health impacts’.

This diversity provides an opportunity to extend the scope of ways that public health research contributes to society from a variety of disciplines and in variety of pathways. Capturing and communicating these mechanisms can enable future researchers learn how to focus their activities for impact to take place, and can help funders of health research to support and promote such activities to their award holders. Our motivation in this paper is to contribute to the scarce evidence base on public health research impact analyses, to demonstrate the value of public health research in its diverse forms, and bring to light the mechanisms and pathways through which public health research contributes to society. Through this we hope to provide accountability and advocacy for the field and inform both researchers and funders on the types of mechanisms, pathways and activities that could be supported to further encourage wider impacts from public health research.

At the same time, we are mindful of the challenges posed by conducting impact analysis for such a diverse field. Generally, the challenges in conducting impact analyses relate to data access, time-lags and non-linearity of impact pathways, and attribution of impacts to research [4–6]. Firstly, the data challenge is that, unlike academic publications, impact data are not currently captured in a systematic way [7]. There are a growing number of tools to facilitate the collection of evidence for impact within research organisations, such as Researchfish® and Symplectic, through which researchers can report their impact related activities, such as media engagements, conferences, non-academic publications, and such. However, there is no standardized mechanism for evaluating or even reporting on impact more broadly (i.e. ‘how did you actually make a difference?’) and many researchers are not yet used to reporting impact regularly. For the purposes of the UK 2014 REF, information was collected in the form of “impact case studies” consisting of roughly four-page narratives now available to read in an online searchable database,^2^ which allowed for a more detailed description of the impact journey and the impacts themselves. Secondly, the timing of capturing impact information is challenging as it can take in the range of 15–25 years on average for research to be translated into public impacts [8]. Furthermore, recent investigations into research impact mechanisms have highlighted that while both researchers and policy makers often assume a linear relationship between academic outputs and corresponding benefits to wider society, the majority of impacts are indirect and consequently very challenging to capture [5, 6, 9]. This literature suggests that many impacts develop over time through formal and informal professional networks and processes. These processes include ‘knowledge mobilisation’ [10], the use of an embedded researcher [11], and ‘co-production’ methods between researchers and practitioners [12]. There is also evidence suggesting that some impacts are achieved during the research process itself, especially in cases where the research was co-created with beneficiaries. Studies such as the Retrosight studies [13, 14], the analysis of the REF 2014 impact case studies [15], and a recent evaluation of the NIHR HTA programme [16], have all highlighted examples of research where study participants had already received the benefits of the research. Analyses of impact therefore often include the intermediary steps, or ‘proxies’ to the ultimate impact. Finally, it is also evident that research projects build on each other in order to reach impacts, which makes it challenging to attribute single impacts to single researchers or projects.

## Methods

### Approach to our study

Against this background, it is important to consider the broad scope of public health research to provide more comprehensive analyses on what occurs in the field. To simply rely on specific types of impact, such as a national change in public health policy, may neglect other impacts on beneficiaries which occur during the research itself. Furthermore, many public health research projects may not follow a linear ‘evidence to public health impact’ pathway; studies have highlighted that research evidence is the least frequently used form of information in public health policy and programme decision-making [17, 18]. These highlight that the context in which public health studies are undertaken, and the information needs of the different roles within organisations, are a crucial part of any examination of impact.

With this in mind, we have approached our study by following the ‘payback model’ by Buxton and Hanney [19], a conceptual framework that has been identified as the dominant one (though not specific to public health) for describing how research leads to impact according to a review by Raftery et al. [20]. This model articulates the stages of research, from conceptualisation to impact, leading to five possible kinds of payback: knowledge production (e.g., academic publications), research targeting and capacity building (e.g., training new researchers), informing policy and product development (e.g., information base for clinical policies), health and health sector benefits (e.g. cost savings and greater equity), and broader economic benefits (e.g., commercial spin-outs). Designed as a method to evaluate the payback for particular pieces of research, the model requires in-depth case studies and an evaluation of both quantitative and qualitative information, including detailed interviews with those who conducted the research and created the impact. We adopt these approaches while mindful of the opportunity to discover other impacts that may occur during any part of the research process.

Given the lack of standardized databases on ‘impact’ alluded to earlier, we have also had to select a source of data for impact based on projects funded by a single funder, the National Institute of Health Research (NIHR). The NIHR funds a variety of health and care research and is one of the main sources of funding in the UK for public health research [21]. The NIHR has two funding streams that explicitly name public health within their titles: the School for Public Health Research (a partnership between eight academic centres with excellence in applied public health research in England) and the NIHR Public Health Research Programme (that provides funding for research on non NHS interventions to improve public health), and we sought to identify projects that included these two funding mechanisms and beyond.

### Aim of this project

Our specific aims were to (a) map the diversity of public health research funded by the NIHR, in terms of funding mechanisms, disciplinary contributions, and public health impacts, (b) through interviews identify examples of impacts, and pathways to impact that existing reporting mechanisms (such as Researchfish)^3^ may not otherwise have captured, and (c) provide illustrations of how public health researchers perceive the generation of non-academic impact from their work.

### Data sources

NIHR established a Public Health Overview (PHO) team, based out of the NIHR Evaluation, Trials and Studies Coordinating Centre, University of Southampton (one of the five NIHR managing centers responsible for delivering NIHR’s operations). The PHO team had already tagged studies funded by the NIHR research programmes and Schools to create a subset of studies pertaining to public health research. This mapping exercise sought to analyse the portfolio since the NIHR’s formation in 2006, and identify gaps in evidence. The outcomes of the mapping exercise have been published for studies funded up to 2013 [21]. The database provided to us for our analysis by the PHO team in early 2017 covers the period 2000-March 2016, and we are aware that the database and its working definitions are continually updated. Table 1 shows the inclusion and criteria used by the team for identifying these studies at the time:

**Table 1:** Inclusion and exclusion criteria for classification of projects as ‘public health research projects’ within the NIHR public health overview (PHO) dataset used for this analysis

Inclusion criteria:	
• Preventative interventions at the population level	
• Early identification and screening programmes	
• Identification of clinical thresholds and care pathways of common conditions	
• Health inequalities	
• Improving services – health needs assessment and health planning	
• Health protection including patient safety, infection control	
Exclusion criteria:	
• Treatment interventions with the exception of treatments for perinatal mental disorders and where increased physical activity is used as the intervention	
• Basic clinical studies	
• Diagnostic test	
• Workforce issues, including leadership and training issues	
• Projects investigating study design	
• Secondary prevention studies (e.g. prevention of relapse of depression)	

We note that since the database was made available to us, the PHO team has published their approach to identify projects that could be tagged as public health research, in which they focused on investigations and/or studies that are anticipated to have “*an effect on health or on health inequity at a population level*.” [22] A total of *n* = 1386 studies were identified from projects funded between 2000 and 2016 as being public health focussed within the PHO dataset. The PHO studies were subsequently categorised according to the Public Health Outcomes Framework [23] to identify the types of outcomes addressed in each. To give us an indication of studies deemed likely to have begun achieving impact, the PHO team identified a subset of projects eligible to go into a Researchfish submission period with PIs whom NIHR was asking to report data. A total of 857 such studies were identified, using a mix of automated and manual matching. All visualisations of project outputs shown in the graphs in this paper are based on either the original *n* = 1386 studies identified or this smaller set of 857 projects provided to us at the time.

### Identifying interviewees and preparing for interviews

From the final dataset received, we selected a range of projects to ensure a mix of sizes and research topics. To do this, we classified each project according to:

*NIHR source of funding:* One project could have been supported from more than one source of funding (Researchfish database allows this information to be captured).

*Size of funding award:* We grouped projects into three sets: £0–£350,000; £350,000–£1million, and; over £1 million;

*Domains of improvement set out in the Public Health Outcomes Framework:* Healthcare public health and preventing premature mortality (which in the dataset was split into ‘healthcare public health’ and ‘preventing premature mortality’ as two separate domains), Health Improvement, Health protection; and Improving the wider determinants of health.

Every project was assigned a funding diversity score depending on how many funding streams supported it (minimum 1, maximum 2 in our database). For each outcome domain we created three sets of projects according to funding size, and within each set we selected the projects with the highest funding diversity score. This meant all scores of 2 were selected and complemented with a random selection of projects with a funding diversity score of 1. This yielded a total of 75 projects. This was our new sample from which to then randomly select projects for interview – during which we checked our list to ensure there was diversity with respect to institutions. In addition, we contacted the NIHR School for Public Health Research directly to ask for suggestions of studies, since they do not report via the Researchfish platform. We then manually identified the different kinds of research designs and methods used in each of our interview sample projects to observe how different approaches influenced the pathways for research impact. Our interview sample spanned both quantitative and qualitative methodologies, including randomised controlled trials, natural experiments, mathematical modelling, systematic reviews, ethnographies, and mixed-methods studies.

We invited a total of twenty PIs to participate in a semi-structured telephone interview, of which ten accepted. One PI was unavailable for the relevant period, and therefore nine case studies were included in our final sample. Table 2 provides a synthesis of each project we investigated. Where possible we conducted follow up interviews with other members of the sampled study teams in order to enrich our understanding of the research process and impact mechanisms. This happened on five occasions, therefore a total of 14 interviews were conducted. All interviews were conducted using a single topic guide (see Additional file 1: Annex A), which addressed the nature of the research project and any collaborators, the findings and mechanisms for sharing these findings, any perceived impact from the project, the perceived mechanisms via which any impact was achieved, and any perceived barriers to achieving impact from research. The categories for impact in this topic guide were inspired by those in the ‘payback model’ by Buxton and Hanney [19]. Interviews lasted approximately 45 mins and were audio recorded with participants’ consent. Interviews were conducted by five members of the project team, with the majority of interviews conducted by HB and AK.

**Table 2:** Case study projects with impact summaries

Project Title	Institution	NIHR Support Stream	Period of support	Funding amount (£000 s)	Summary of impact
How effective is the Forestry Commission Scotland’s woodland improvement programme - ‘Woods In and Around Towns’ (WIAT) - at improving psychological wellbeing in deprived communities?	University of Edinburgh	Public Health Research (PHR) Programme	2012–16	350–1000	This study set out to explore links between environmental interventions – in this case, efforts to regenerate and enhance access to local woodland – and local residents’ perceived stress and mental wellbeing. The principal investigator, a professor of landscape architecture, worked collaboratively to generate ideas for the research from an initial concept developed with the Forestry Commission. The multidisciplinary research team included epidemiologists, health geographers, psychologists, and health economists. The team had not yet published findings, but noted a number of practitioner groups, as well as membership of a NICE guideline-writing group on physical activity, as receptive audiences with whom they had developed functional links.
Improving employment outcomes for young people with first episode psychosis	South London and Maudsley NHS Foundation Trust	Research for Patient Benefit (RfPB) Programme	2008–12	0–350	This was the first UK randomised controlled trial exploring how to overcome barriers to helping young people with psychosis return to work via the delivery of individual placement and support (IPS) services – an intervention for which there was already good evidence of patient benefit. It examined the effect of training clinically-based vocational staff in motivational interviewing techniques, to enhance their provision of IPS services. The team partnered with ImROC, a non-profit consultancy with experience of working with NHS Trusts to implement recovery-based models of service delivery. Key factors in the success of the study appeared to be the provision of a dedicated member of the ImROC team working with clinical staff to ensure fidelity to the recovery-based model of care. The team also reflected that clinical teams responded positively to seeing the results of patients’ return to work (i.e. social outcomes), helping to overcome a perceived risk that patients were more likely to relapse. The study showed the benefits of placing and training specialist vocational support staff within the clinical team, with significantly more patients remaining employed following their receipt of the enhanced IPS model. The researchers noted a short-term view of the financial burden placed on NHS trusts from employing these staff as a barrier to achieving impact.
An evaluation of a water fluoridation scheme in Cumbria	St George’s, University of London	Public Health Research (PHR) Programme	2013–20	1000+	This study was set up in response to a systematic review and MRC working group report calling for improved evidence of the benefits of water fluoridisation. It seeks to evaluate the effects of reintroducing fluoridisation to a population of 5–11 year-olds in Cumbria, following its removal for a period of 5 years while the water system was updated, and the effect of this on children’s dental caries. The team worked with local schools to recruit participants to the study, reflecting that they held back on using social media as an adjunct to recruitment, due to the negative reaction of groups advocating against fluoridisation. They noted the complexity of convening multiple groups – researchers, commissioners, policymakers – as a challenge for public health research, made easier by having Public Health England as a central convening body. They also highlighted a capacity issue arising from limited clinical staff choosing public health as a career, and the need for advocacy from NIHR to make researchers more aware of the different pathways available to them. The team published their study protocol in 2016, and were awaiting results of the study before publishing further information.
Diabetes Prevention Model	University of Sheffield	School for Public Health Research (SPHR)	2014	Not available	The central aim of this project was to develop a new model to evaluate the cost-effectiveness of public health activities to prevent diabetes. The team had early engagement with representatives from NHS England, who in 2015 had expressed an interest in understanding the economic impacts of activities (such as education on healthy eating and lifestyle, help to lose weight and bespoke physical exercise programmes) planned under the national Diabetes Prevention Programme. The team developed the model into an online tool that allowed local NHS commissioners to assess the potential return on investments from prevention activities planned in their local areas, and on different local populations. In parallel they also developed a similar tool for local authorities, following a call from Public Health England. Information from the modelling work informed rollout of the Diabetes Prevention Programme in 2016 and work commissioned by NICE on who to prioritise when planning diabetes prevention programmes.
TOMMY trial: A comparison of TOMosynthesis with digital MammographY in the UK NHS Breast Screening Programme	University of Cambridge	Health Technology Assessment (HTA) Programme	2010–13	1000+	Breast tomosynthesis is a recently-developed technique that uses a series of low-dose X-rays to build up a 3D picture of the breast tissue. The team’s motivation for trialing this technique was to detect cancers when they were smaller, and reduce the ‘recall’ (false positive) rate of patients called back for further testing. The team worked with INVOLVE and a patient advocacy group – Independent Cancer Patients’ Voice – on the best way to design an initial trial, which recruited to its target of over 7000 women and was published in 2015. Discussions with Public Health England and the National Screening Committee made it clear to the study team that this initial data was insufficient to effect a policy change on national screening for breast cancer. They highlighted both the conservative nature of these organisations, and also the controversy of breast screening with the public. The team are therefore using the data from the initial trial to design a larger trial involving 100,000 patients. They are considering ways to involve policymakers upfront, and also working with a social scientist to explore public perceptions of screening in the context of their research.
A 1-year follow-on study from a randomised, head-to-head, multicentre, open-label study of two pandemic influenza vaccines in children	University of Oxford	Health Technology Assessment (HTA) Programme	2010–11	350–1000	This study responded to the UK Government’s purchase in 2009 of two pandemic influenza (H1N1, or ‘swine flu’) vaccines, Celvapan and Pandemrix, and an urgent need to test their safety and efficacy in children. Public Health England had contacted the Oxford group given their ‘track record in delivering trials’, though the study team involved three other centres with established clinical trials groups. The team fed emerging results through to Public Health England and the Joint Committee on Vaccination and Immunisation, as data was gathered. They reflected that the UK was unusual in using unpublished data in this fashion to inform policy decisions, and noted that the UK regulatory environment and exiting relationships with the Department of Health helped ensure that the research was able to move swiftly, given the emergency nature of the pandemic. Impacts of the trial included the children who were vaccinated and the Department of Health, who benefitted from guidance on how to conduct trials in pandemic situations. One negative impact was an association with children developing narcolepsy, which was noted as a rare side effect of the Pandemrix vaccine.
Evaluation of a National Surveillance System for mortality alerts	Imperial College London	Health Services & Delivery Research (HS&DR) Programme	2014–16	350–1000	Following their work on the Shipman Inquiry, the Dr. Foster unit at Imperial College London developed a national system to generate monthly alerts from routinely collected hospital administrative data on 122 diagnoses and procedures. Their evaluation was designed to address concerns of bias and establish the extent to which variations in quality of care was a factor in triggering alerts. The team had already been sending mortality data freely to NHS trusts, copying the Care Quality Commission (CQC) if it exceeded a certain threshold. They ran a workshop with CQC to help with recommendations based on their research, in particular the service and mode of delivery of the alerts. The team were taking a mixed-methods approach to understand institutional responses to mortality alerts. While they noted that the research was at an early stage, and that death is a crude indicator of underlying issues, the team hoped that their approach was supporting best practice in CQC investigations, and how hospitals can respond well. They flagged that social media engagement around their findings had ‘turned nasty’ due to its timings coinciding with the junior doctors’ strike.
Comprehensive Longitudinal Assessment of Salford Integrated Care (CLASSIC): a study of the implementation and effectiveness of a new model of care for long-term conditions	Salford Royal NHS Foundation Trust	Health Services & Delivery Research (HS&DR) Programme	2014–2016	1000+	The aim of this project was to evaluate the changes that had been made in Salford to the provision of care for long-term conditions reflecting a move towards integrated care. The study found that integrated care did not have a significant impact in terms of reducing hospital admissions as intended. The challenge in disseminating these findings since they are in contradiction to the current direction of travel in terms of the organisation of care within the NHS and the wider policy landscape. Significant investment has been made in integrated care in some locations and as such this is a challenging message to share that will not necessarily be openly received by policy makers and practitioners.
Prenatal screening and treatment strategies to prevent group B streptococcal (GBS) and other bacterial infections in early infancy: cost effectiveness and expected value of information analyses	University College London	Health Technology Assessment (HTA) Programme	2005–6	0–350,000	HTA commissioned this study in the context of wider decision-making processes around levels of evidence required to adopt screening strategies for common infections in early infancy. A larger randomised controlled trial costing £12million had been planned, involving 600,000 women, to evaluate the effect of screening for group B streptococcal (GBS) infections. The 2005 study brought together methods experts in techniques to model the economic value of generating further evidence – in this case, applied to existing treatment and screening options for GBS infections. The main impact was that the study team concluded that running the larger controlled trial would not be cost-effective: the trial was therefore called off. Instead, resources would be better spent on treating patients at a higher risk of infection, and investing in developing a vaccine. The team noted that their involvement in fora that brought together different groups throughout the research – their case, a patient advocacy group, members of the larger planned trial team, and the UK National Screening Committee – provided the means to allow them to apply relevant evidence from their study. They noted a barrier to further impacts in this area as the challenge of making routinely captured infection surveillance (i.e. audit) data available for research purposes, and that trials funding might usefully be redirected into ways of improving access to and use of these data.

### Analysis and synthesis

Using insights from the quantitative analysis and interviews, the project team conducted two workshops to explore the emerging findings: one with four invited participants including Directors of various NIHR research programmes and representation from the NIHR Public Health Overview function, and an internal analysis workshop during which the project team discussed the case studies in depth, addressing the nature of impact in public health, impact mechanisms and pathways, and the way in which impact was understood by PIs. Our analysis was conducted in the tradition of grounded theory, establishing core themes through an open coding process and populating these with corresponding interview data. Analysis was conducted by HB, SHK, SG, AK, IG and BI. Any differences in interpretation were resolved through discussion among the project team.

### Caveats

One major limitation of our study is that we have focused on one UK-based funder only. This could potentially limit our understanding of impact within public health, as we are not drawing on the full extent of research across the country (or indeed globally). As noted earlier, however, the NIHR is one of the main sources of funding in the UK for public health research. Furthermore, the NIHR does provide a varied sample of research projects relating to public health because of the breadth of programmes, methods and health areas that it funds. While our case studies are drawn from one funder, our findings should be interesting to those examining research impact more broadly, both in the field of public health and beyond. We do not claim to showcase a representative sample of public health research projects in the UK, but a sample that illustrates researchers’ experiences and perceptions of the varied mechanisms that demonstrate how impact is created from public health research.

We are also mindful that this article is written from the perspective of how we define public health in the UK. Although the UK is found to be one of the main contributors to publications classed as public health within Europe [2], we are mindful that other countries outside of Europe may have different scope and definitions they employ. As noted in the Background section, however, most definitions imply a focus on health of the population, which is what constituted the focus of the PHO criteria for identifying the projects tagged as public health made available to us. We have relied on the NIHR PHO coding of projects to identify our sample, from which we selected our interviewees. We trust this process was completed rigorously although caveat that we may have missed some examples of projects that could also have been considered public health. However, we feel that because we are not making general claims about public health research as a whole, nor of the totality of research in the UK, this limitation does not have a significant impact on our conclusions.

## Results

Our focus in this paper is to illustrate the perceptions and experiences of public health researchers in demonstrating and delivering on non-academic impact from their work. This is captured in section (ii) of our results. First, to provide some background and overview of the public health research portfolio at the NIHR, in section (i) we describe the general trends observed from the full dataset we received of projects that were reported on Researchfish and were tagged as public health projects by the NIHR team.

### Mapping the funding mechanisms and impacts for public health research

In this section we describe the general trends observed by mapping the data from the projects we received (those that had data available within the Researchfish reporting dataset).

#### A variety of funding mechanisms support public health research

**Fig. 1:**
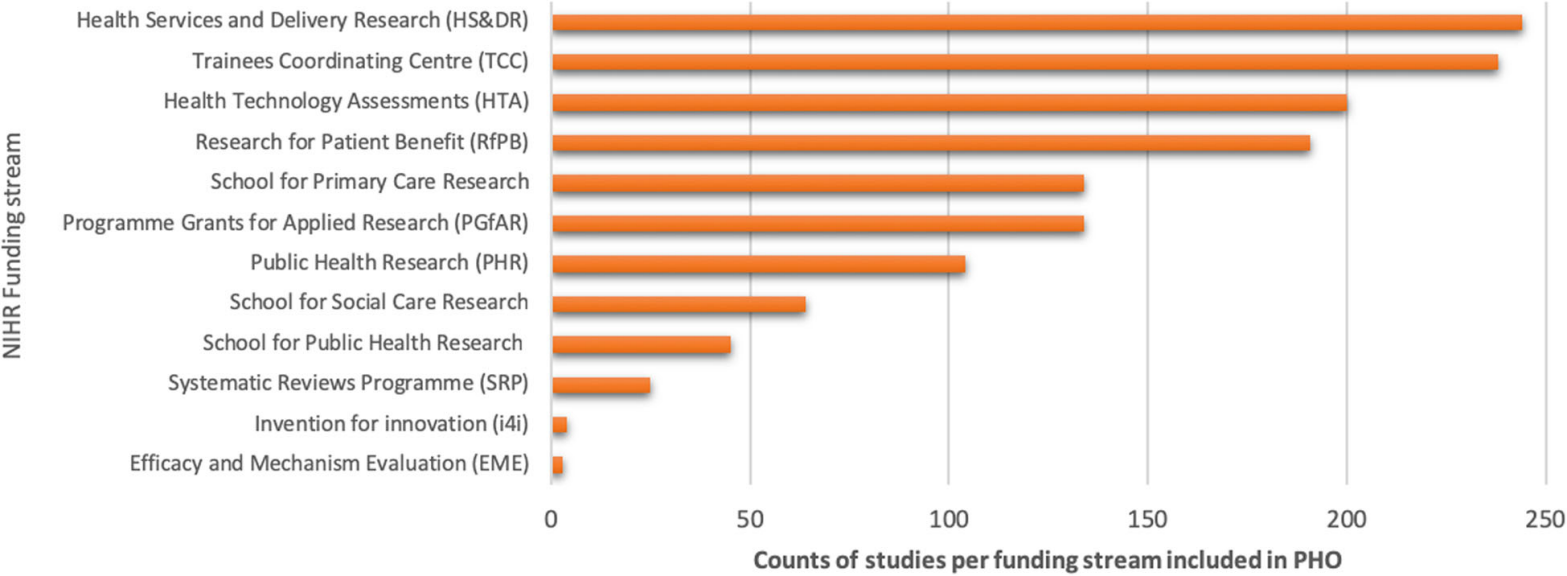
Counts of studies included in the NIHR Public Health Overview (PHO) dataset received from our analysis, by their respective NIHR funding stream (total *n* = 1386)*. * Note: The NIHR has different managing agents for coordinating funding and research delivery. The funding streams included in this diagram run across different managing agents. The exception is the Trainees Coordinating Centre, for which we have grouped all their projects into one as they are training schemes and are broken down by stage of career, and this would not indicate the type of diversity we are illustrating here in terms the types of funding provided

As noted above, we identified 1386 projects funded between 2000 and 2016 as being public health focussed for our analysis. We mapped the funding streams for each of these projects and show these in Fig. 1 and note that there are many more funding mechanisms within NIHR that fund public health research activity (Fig. 1). In addition to the NIHR School for Public Health Research and the NIHR Public Health Research Programme, 89% of the 1386 projects are funded via other funding programmes, demonstrating the diversity of funding streams that support public health-related research. A large proportion of these are funded through the Health Services and Delivery Programme (HS&DR). It is worth noting that several of the large funding streams (e.g. the NIHR HTA programme) support a significant number of projects that are classified as public health (Fig. 1). Although this may be due, in part, to the length of time that some of the programmes have been running, this chart captures the diversity of ‘public health’ and the range of types of research that can contribute to public health outcomes.

#### A variety of impacts arise from public health research

**Fig. 2:**
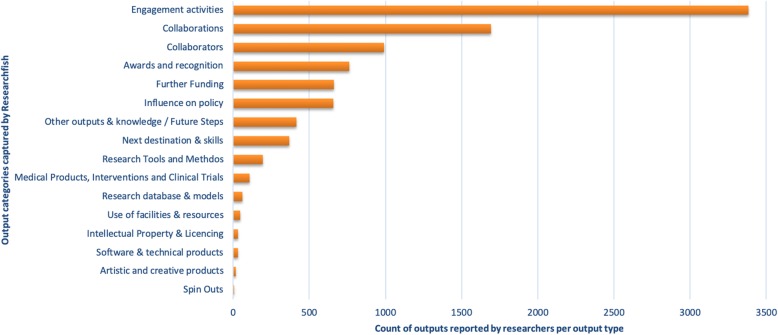
Counts of research outputs self-reported by principal investigators using Researchfish, by Researchfish output category, excluding academic publications (n=9428 reported activities in the 857 projects included)

We also mapped the types of impacts reported by researchers in the dataset received. Within the Researchfish online interface, all individual entries are labelled as ‘outputs’ of research, including academic and non-academic outputs and any wider outcomes that may be considered ‘impact’, entered by researchers themselves. Looking at this data self-reported by researchers via the Researchfish platform – a subset of 857 studies matched within the NIHR Public Health Overview portfolio – we see a diverse range of different types of research activities and outputs (Fig. 2). In line with previous analyses of data captured via the Researchfish platform, we found that investigators reported academic publications more frequently than any other output category. Following publications (not included in the table), ‘engagement activities’ is the most commonly reported item in Researchfish (3383 instances), followed by ‘collaborations’ (1692). However, we also note a smaller but still substantial number of impacts on patients (724), and policy and practice (658). We note that these graphs rely on data self-reported by researchers, and therefore the emphasis given to particular types of research activity may determine what was reported within Researchfish. Each researcher may also have interpreted their activity different (putting an entry under ‘engagement activity’ which someone else may have considered to be ‘policy and practice’ or ‘collaborations’). Through our qualitative interviews, the findings of which are described in section (ii), we were keen to identify what these activities, such as ‘engagement activities’ or ‘collaborations’ entailed.

### Researchers’ perspectives on pathways and mechanisms to impact

In this section we report on the findings from the qualitative interviews, focussing on researchers’ experiences in producing and articulating the impact from their work.

#### Different interpretations of the meaning of impact from public health research

The diversity we observed in the quantitative data was reflected in our in-depth case studies. We found that researchers had different interpretations of what public health could include as a research discipline, and several of those we contacted questioned whether their research should be classified as public health at all.

One of the main themes arising from our interviews was diverse interpretations of how evidence created from public health research can lead to impacts in national policy. Interviewees felt that the diverse forms of evidence produced by public health research do not always correspond with those required by policy makers to effect change. For larger, drug-based interventions, randomized controlled trial methodologies may still generate the most appropriate form of evidence, but this is not the case for many interventions in public health, especially those related to lifestyle factors. Our participants reported lack of clarity as to what constitutes appropriate evidence to achieve impact. As one researcher noted, *“there are different expectations for different fields about what counts as robust evidence. You could argue that you are unlikely to do harm in a community by making their park easier to use, but we are held to same standard as clinical drug trials. You can’t easily do RCTs in this field, but that’s the standard to which health evidence is held.*” *Interviewee 1.*

We also found that researchers struggled to ascertain clarity on the level of evidence required to influence public health policy. Having worked on a study that was deemed by a national committee not to have produced sufficient evidence to bring about a policy change, one interviewee told us that they had subsequently asked what would be needed to achieve this, but were not given a tangible answer. Reflecting on this, the researcher commented *“[policymakers] are understandably cautious … so it makes it difficult for an academic to know how much evidence you need in order to actually effect the change” Interviewee 2.* For this researcher, the uncertainty surrounding the level of evidence required left them feeling that it was a struggle to make changes in a timely way.

The uncertainty regarding the type of evidence required for change was a recurring theme emerging from our discussions with researchers, one of whom had specifically developed techniques to model the economic value of generating further evidence regarding screening and treatment strategies to prevent infections in early infancy. Reflecting on how to tease out whether and how further evidence was needed, the researcher explained:

*“‘What do you want to do now, given the evidence such as it is?’ and, ‘Do you need more evidence to inform that choice in the future?’ By separating those two questions out, you can have a sensible answer to both, to completely move away from hypothesis testing.” Interviewee 3*

Reflecting on the way researchers discussed national policy impact, we noted that their responses often suggested that their main interpretation of what ‘impact’ means is evidence that produces change in national policy. When interviewed, a few of the researchers were cautious in describing the impact of their projects, emphasising that they had not quite produced all the evidence to reach policy. Yet when probing further we found that small, unintended benefits from their work was directly observed with the non-academic organisations with whom they worked, such as health service delivery or local government. When describing the relationships developed during research projects (described further in the next section), they noted that these relationships themselves had the capacity to influence change directly within an organisation. For example, one researcher commented that while they considered the potential primary impact of their research to occur at a national level, the more immediate benefits occurring at a local level were the “*unintended impact.*” *Interviewee 4.*

The challenges in getting evidence into policy and practice apply to both national and local contexts, as pointed out by several of the researchers we interviewed (whose perspectives are from the UK contexts). Reflecting on the devolved nature of public health in the UK, one participant told us:

“*The problem is that the research has been rather dissociated from the practitioners … Cost effectiveness, timing, relevance and generalisability have been rather lost … We’re learning how to work with local government, making relationships … We’d done all that over 50 years with the NHS, and now we’ve got to do it with local government.” Interviewee 5*

In spite of enthusiasm for interdisciplinary collaborations, uptake in practice is not always possible due to the silos in the way health, and other services that support health, are delivered throughout the country. One interviewee referred to the lack of cross-departmental collaboration on public health issues:

*“We have multidisciplinary/interdisciplinary research findings, so to achieve practice based on them you need intersectoral budget management to reflect delivery demands. I don’t see any sign of that happening.” Interviewee 1*

The responses in this section suggest that there are different interpretations of what constitutes evidence required for public health research impact, especially if the impact is intended to occur at national policy level. Smaller, localised benefits from public health research are also acknowledged, although not always interpreted as what counts as ‘impact’ by the researchers.

### Engaging external stakeholders to facilitate impact

Following academic publications, engagement activities were the most commonly reported item in the Researchfish outcomes data we received in our dataset (Fig. 2). Our interviews enabled us to explore the nature of these activities. One of the most significant impact mechanisms reported by our interviewees was the relationships they developed with a range of external stakeholders, including hospital trusts, the Department of Health and the medical technology industry. Several of our participants suggested that these relationships were a way to navigate the complexities of the public health landscape. We noted that these relationships appeared to be most effective if they were in place from the start of the research, and several of our interviewees reported calling on relationships with external stakeholders that they had known professionally for many years.

One researcher informed us that their team had been chosen by the Department of Health to respond to a public health emergency on the basis that the researchers and their work were already known and trusted by those in a position to implement their findings. This researcher also noted that good relations between the Joint Committee on Vaccination and Immunisation (JCVI) and researchers meant that research findings were able to inform practice much sooner:

“*There are good links between academia and JCVI which makes the UK well-positioned in being able to access the necessary data quickly in order to inform critical decisions without having to wait for things to be published.” Interviewee 6*

As a counterpoint to navigating through the intersectoral silos identified in the previous section, a selection of interviews demonstrated initiative in reaching out to organisations outside the health sector, pointing out that they could be the people to help facilitate impacts over the lifetime of the research study and beyond:

*“If you want something done about the environment, you have to be working with parks managers in local authorities and organisations like the Forestry Commission or the National Trust - people who give grants and who are going to make physical difference to real environment - if you want your research to inform policy and practice.” Interviewee 1*

Similarly, another one of our participants emphasized the importance of generating the necessary relationships with a broad range of external stakeholders to garner the necessary support for a new health initiative:

“*You have to set it up with the policy makers first, and then the funders, do the work and gather the evidence to make the change. I wouldn’t go directly to NIHR unless I had support from the screening committee of England. You need to lay the groundwork. That’s one way to impact, getting opinion leaders on board, getting the community behind you and the professional bodies and policy makers”. Interviewee 2*

Several of the researchers we interviewed pointed out the importance of these relationships in facilitating hitting the right policy or impact ‘window’, sharing examples of where timing had been a principle factor for both policy-makers and practitioners. One researcher expressed concerns that findings were not informing practice in time to be useful to practitioners:

*“Practitioners are completely uninterested in research findings in 5 years’ time.” Interviewee 3*

Although they emphasized the need to reduce the time it takes for research findings to reach practitioners, we also found that research that is ‘ahead of the curve’ may not attract interest from policy makers until years later, particularly if these relationships are not already in place. Another researcher told us that although their project initially failed to gain the necessary support in PHE to effect the desired change in national screening guidelines, they were approached years later and asked to contribute their expertise on the screening technique their team had advocated, at a time when the need was felt to be greater and the benefits of the technology better understood. Here we see a different scenario, in which researchers had to wait for a ‘policy window’ years down the line:

*“Interestingly, PHE have now realized they have a real manpower problem and so have come back to me nine years after I published evidence on reading screening mammograms. Sometimes it’s about timing. Maybe we were ahead of the game at that point … it’s about timeliness.” Interviewee 2*

While generating relationships with external stakeholders was often rewarding, researchers also commented on how this can take time and is “*resource intensive.” Interviewee 7.* It can therefore be frustrating when there is high turnover either in policy or hospital practice so that personal relationships with relevant organisations are lost or have to be rebuilt over time. The challenge in building relationships or indeed in engagement activities over and above writing and disseminating an academic paper, is that these usually need to continue after a funded project is closed, and so finding the resources to sustain them is tricky.

### Dissemination mechanisms to facilitate impact

For each of the in-depth case studies, we found that researchers employed a variety of different dissemination activities beyond academic publications. Our participants provided detailed perspectives on these activities, and these discussions suggested that many of the presentations were made to non-academic audiences using infographics, animation and web-based media to communicate main headlines clearly, or engaging with mainstream media as appropriate. Messages and mechanisms were often tailored to those in a position to drive implementation forward:

*“It is not science sitting in isolation; it’s science sitting in a complex group of stakeholders. The pure science piece is the submission to NIHR. But how it is disseminated and spread will have to be very carefully undertaken.” Interviewee 8*

Several of our researchers highlighted mainstream media as a means to facilitate impact, offering examples of where a news story had helped drive change. One researcher told us:

*“I’ve had experience of high profile bits of work which have effected policy change, and that has involved a lot of exposure on the media, you know, almost putting pressure on the policy makers to do something about it.*” *Interviewee 9*

Another researcher told us that they had given an interview about a high-profile research project for a popular monthly magazine, and subsequently received a telephone call late at night from a patient overseas who wanted to discuss the findings after having read about the study in the magazine.

Being mindful of the right communication channels also meant not investing in activities that were not appropriate for a particular project, for example, not soliciting mainstream media if the individual conversations and meetings with stakeholders were more important and would help drive the adoption in practice. One researcher told us:

*“We were directly addressing those to the audiences that needed to hear them, either through presentations or the reports. So we stopped there, and actually I think that’s appropriate. I think those messages needed to be agreed with and then owned by others in order to take them forwards. I think it would have been inappropriate for us to be pushing [media engagement].” Interviewee 10*

Several researchers also noted that engaging with social media could be challenging, with some having had negative experiences which had made them more cautious about this kind of dissemination mechanism:

*“It’s a jungle out there. It all got very nasty very quickly, and actually my poor junior researcher who happened to be corresponding author and the first author on the paper, he had the most awful time with Facebook campaigns against him, and it was a really nasty business.” Interviewee 9*

Overall we observed a balanced approach on the part of most researchers to selecting and engaging with different dissemination mechanisms. However, when reflecting on their experiences with media engagement, several researchers noted that bad experiences with both mainstream media and social media would make them more cautious about using these mechanisms in future.

### Acknowledging “negative” findings

Several of our case studies revealed the difficulties associated with achieving impact with a negative research finding, especially if findings ran contrary to current thinking and practice. As one researcher explained:

*“Positive results can hold attention and enthusiasm because they can be scaled up [ …] so that’s in your interest as an academic. Negative results are harder [ …] This is a particularly difficult space for research for evaluating someone’s innovation—the air can go out of the room.” Interviewee 7*

The same researcher discussed the wider culture surrounding positive and negative findings in terms of research impact, emphasizing the danger of incentivizing positive results:

*“If you want impact, you need positive results, and that’s dangerous for research [...]. Having these incentive structures puts academics in a difficult situation: you need something new and exciting that works, and that can’t or doesn’t always happen.”*

The potential value of impact derived from negative findings was illustrated by another of our case studies, where researchers were able to implement their negative findings successfully. This project used a multi-parameter evidence synthesis to examine the value of screening as one of a number of interventions to prevent infection in early infancy. The study findings indicated that conducting a larger cluster randomized controlled trial to evaluate the efficacy of screening, planned at a cost of £12 million, would not be worthwhile. The researchers commented that:

“*The single most specific impact of the study was to stop the larger cluster randomised controlled trial from going ahead*.” *Interviewee 10*

This saved a significant amount of money, which could then be invested in other public health research projects.

### Researchers as drivers of impact

One of the most striking elements to emerge from our case studies was the role of researchers’ own perceptions and skills in determining research impact. We found a range of different opinions among our participants of when and how impact is achieved, with some emphasizing the role of presentations and collaborations, and others suggesting that the publication of research findings in academic journals was the main springboard for impact. One researcher noted that impact occurs once findings have been written up:

*“Once the organizational, institutional stuff has been properly written up we can then put in some recommendations or guidelines…That’s the plan, but we haven’t got there yet.” Interviewee 9*

Other interviewees highlighted the impacts that occur earlier in the research process, with one noting that participants in their trial benefitted directly from the research in addition to the longer term impacts that they were aiming to have at a national level:

*“The main beneficiaries at the time were the individuals who were vaccinated in the trial, and then more broadly it was the availability of the data to guide the department of health on how to move forwards.” Interviewee 6*

We also observed a number of different views on the drivers of impact from the perspective of researchers. One researcher highlighted passion as a key factor motivating public health researchers:

*“Ultimately, we are not just curious. I come from a discipline where we plan to make a difference in people’s lives in practical ways.” Interviewee 1*

Another commented that the most effective means to achieve research impact was through conducting research that was addressing important public health challenges that are of particular interest to policy makers, the media and the public in addition to the academic community:

*“I think [effecting change is about] just having some interesting research. You know, something that other people can relate to and it’s important.” Interviewee 9*

Some researchers felt duty-bound to ensure that they facilitated impact from their research. As one researcher commented:

*“Retreat if you did a bad study, but if you did a good study, then it’s your responsibility to push that out.” Interviewee 8*

However, while researchers felt that it was important that their work be disseminated in ways that would effect positive change, some raised questions as to how far researchers should be responsible for impact. One of our workshop participants noted the potential for conflict of interest if researchers felt the need to advocate impacts from their own research, and suggested that a neutral third party could take on the responsibility for advocacy.

Similarly, one of our interviewees commented that although researchers should make their findings clear to those with the capacity to implement change, they felt that researchers should stay removed from decision-making, and did not necessarily have the skills to engage in impact activities:

*“I wouldn’t expect impact to be straightforward or simple. I’m not entirely sure I’m skilled enough for that or that it’s my job. Not to say it’s not an important role or responsibility. I feel our role there is to be available, accessible and clear about what we found. In terms of decision making, that is several steps away from me and that’s the way it should be I think. They are responsible. They have to go to their local elected representatives.” Interviewee 7*

We noted that there was also an acknowledgement that the skills required for engagement beyond academic peers, be it with social media, mainstream media, or indeed other forms of communication, are not always readily available to researchers. As one researcher commented:

*“We have capacity issues in public health researchers, particularly those with clinical qualifications.” Interviewee 8*

Our conversations with researchers suggested that they would benefit from support for impact and engagement, both in terms of building skills, and also through building impact elements more explicitly into the research process. One interviewee explained that researchers are not always taught about impact and how to effect change, and suggested that one way to support them in doing that would be to make impact elements a feature of funding applications:

*“We’ve very good at teaching people research methodology, but we’re not very good at teaching them how to influence…In the funding applications… it could be not only have we discussed this with patients and the public but we have discussed this with policy holders, and we have checked that if this is successful then this is what would need to be done. I don’t want to put another barrier in to people getting research money, but it could be useful in getting people to think…” Interviewee 2*

## Discussion

This analysis of the 857 completed or ongoing NIHR-funded public health research projects, which included nine in-depth case studies, suggests that public health research as a discipline can contribute substantially to impact beyond academia. The funding mechanisms that support this broad range of public health research (for the NIHR) extend beyond those that include public health in their title. The pathways to impact observed in our in-depth case studies include contributing to debates on what constitutes appropriate evidence for national policy change, acknowledging local ‘unintended’ impacts, building trusted relationships with stakeholders across health and non-health sectors and actors, collaborating with local authorities, and using non-academic dissemination channels. While this study was by no means exhaustive, there are lessons regarding impact mechanisms and pathways that are useful to draw out for further discussion, that could benefit funders of public health research, researchers, policymakers and practitioners.

### Considerations for public health research institutions and funders

The mapping of the NIHR projects with a public health focus showed that these impacts are derived from a variety of funding programmes offered by NIHR, beyond the Public Health Research Programme and School for Public Health Research (see Fig. 1). This breadth of NIHR public health research is one of the drivers for PHO activity and serves as strong advocacy for the field. While we have not tested this for other funders, it is likely that a broader consideration of the many funding streams that contribute to public health research would demonstrate for other funders the substantial investment and contributions made within the discipline.

During our analysis we reflected that supporting researchers’ initiatives in implementing their pathways to impact should be a principal consideration for funders and supporters of public health research. The researchers we interviewed showed great enthusiasm and motivation for responding to public health challenges. The motivation for having societal benefit (or ‘impact’) from their research is therefore implicit, but we identified difficulties in having the time, financial resources, and skills to take the necessary actions to facilitate it. As Redman et al. [24] have pointed out, while there are courses, tools and funding modules to assist researchers in making an impact, there is little evidence about whether these approaches work in practice, and more could be done to support researchers in making the connections they require to facilitate impact. It is not to say investigators should play no part in enabling impact from their work; indeed, some of the researchers we interviewed showed initiative in engaging stakeholders outside of their field and taking part in non-academic dissemination of their work. For others, however, there needs to be an acknowledgement of the skills and resources required for engagement – or more nuanced impact-facilitating – activities, and these may have to be shared with other colleagues or support staff.

Our qualitative findings suggest that relationships with those in a position to facilitate change were developed and strengthened over time, providing short, medium and long-term impacts that helped facilitate public health goals. Greenhalgh and Fahy [6] observed examples of this in their discussion of 162 REF case studies, commenting that “In the policy setting, [impact] occurs when researchers and policymakers, through repeated interaction over time, come to better understand each other’s worlds and develop goals that are compatible if not fully aligned.” In their work on ‘productive interactions’ Spaapen and van Drooge focus on capturing information about knowledge exchange interactions between researchers and stakeholders as a prerequisite to achieving impact [25]. Our case studies yielded some examples where relationships with policy-making bodies had been developed over years, and others where impacts had emerged from more fledgling relationships with stakeholders, for example through researcher interactions with hospital staff. Indeed the devolved nature of public health in the UK (following reforms in 2013 in shifting public health services and planning to local authorities) requires an understanding of local public health challenges in addition to wider national policy requirements. A systematic scoping review of the use of evidence in local English public health decision-making identified the primacy of local evidence, the important role of local experts in providing evidence and knowledge, and the high value placed on local evaluation evidence despite varying methodological rigour [26]. This suggests the need for supporting relationship building and engagement between public health researchers/experts and local decision-makers to create localised impacts. In another study data drawn from an NIHR-funded project examined how evidence is used in commissioning and planning, and also concluded the role of localised knowledge. They found that published evidence is “made fit” for local commission and planning by relating the evidence to local context and needs and tailored into local actionable messages [27].

How support for such long-term engagement is put into practice can vary for funders or research institutions – some resources may be available within universities in the form of ‘impact officers’ or research administrators. There is also much to be learnt from ‘translational research’ or guidance for public health organisations on how to develop ‘knowledge to action’ strategies such as fostering connections among stakeholders, and working beyond their academic institutions to understand contextual factors [28]. Furthermore, there are other models for facilitating uptake of research. Ward [10] provides a useful framework for identifying and making use of ‘knowledge mobilisers’ who can facilitate getting evidence into practice or policy. The use of an ‘embedded researcher’ has been shown to be productive also in mobilising knowledge in general quality improvement [11] but also specifically in exchanging knowledge between public health research and a local authority in the UK [29]. Another mechanism is the use of the researcher in residence model [30]. All of these do require either shifting of funding resources on existing projects or funding specific for these types of activities.

Deriving maximum benefit from such relationships requires not only institutional frameworks that support stakeholder engagement, but also building the skills and confidence of researchers in their engagement activities and building trusted relationships. Our findings suggest while it is important that stakeholder relationships are established through research, these relationships are far more effective if they are maintained and strengthened over time. Upton et al. studied individual and institutional perspectives on research impact in nine universities and found that qualities to enable impact include a high degree of motivation with regards to knowledge transfer mechanisms and other skills that support translation [31]. Bayley and Phipps [32] argue for building ‘impact literacy’ - developing staff capacity to judge, articulate and optimise impact across contexts. They highlight evidence that integrated methods of generating impact are more effective than end-of-grant methods. It is also important to provide academics with opportunities to develop their networking, media-management and advocacy skills in order to ensure that they can take best advantage of opportunities for stakeholder engagement.

Finally, our study suggests that funders can also work collaboratively with researchers to facilitate impact, through incorporating consideration of impact more fully into research planning and upstream assessment of applications. Funders could also support early and frequent collaboration with stakeholders, targeted engagement activities, and dialogue with award holders on appropriate downstream evaluation of impact, throughout the research process. Though evidence of the causal nature of this is not conclusive, Guthrie et al. [13] showed that collaborations between different groups (industry, patients and practitioners, regulators) was associated with impact across a number of domains. Researchers can also be supported in maintaining their impact activities through their funder’s existing mechanisms, such as NIHR’s existing research funding ecosystem, which provides different opportunities for dissemination and encourages participatory research and patient and public involvement in research.

### Considerations for public health policy and practice

Our analysis suggests that the pathways and activities that lead to wider impact are diverse, and can occur both during the research process and on a longer-term basis. We observed more traditional forms of impact, including research that had influenced policies or guidelines (especially those reported by researchers within the Researchfish database), but also less tangible impacts such as research that empowered project participants to effect change in the hospital trusts in which they worked. These findings have implications for how public health research can influence public policy and practice in three ways: the nature of evidence and evaluation, the need to engage with local authorities, and importance of cross-sectoral working.

One of our most striking findings is that, while impact is often conceptualised in terms of having impact at a policy level, there are also many forms of impact that can emerge in localized settings and numerous different environments. It is also the case that where research might be best utilized to improve public health policies and guidelines, the evidence produced is by no means guaranteed to reach those who have the power to effect change such as this. As Hunter [33] points out, while most public health research is government funded, the extent to which research findings are used to inform policy are extremely variable. Macintyre [34] argues that both policy makers and researchers need to develop their understanding of impact mechanisms further, to ensure that public health research supports improvements in public health:

*“Policy makers certainly need to be more sophisticated in understanding and commissioning different types of research and acting on it. However, researchers also need to be much more sophisticated and less naive in understanding how research does and does not influence policy, and how to go about helping policy makers to interpret the jigsaw of evidence, and its relevance and usability”.*

Our study also highlights the importance of acknowledging the context in which evidence in public health is deployed. Case studies touched on the need to think about timing (hitting the right ‘policy window’ as suggested by Kingdon [35] and discussed by Cairney [36]), negotiating the presentation of negative findings, and understanding the different types of evidence required by those responsible for implementing public health initiatives. It may also be the case that the type of evidence required for change goes beyond that traditionally produced by researchers, which would also require a greater focus on how results might translate in specific social and political contexts. For example, in her evaluation of the kinds of evidence used in understanding the social determinants of health, O’Campo argues that “research should not stop at demonstrating whether and how a program or policy improves well-being. More detailed information is also needed to facilitate adaptation, tailoring, and implementation of those programs and policies to local settings and target populations” [37]. Further, while evidence drawn from large controlled intervention trials meets higher standards of rigour, this evidence is not necessarily the most applicable to ‘real world’ practice [38] and may hinder the implementation of cost-effective interventions. Fischer et al. have argued that in the case of many public health interventions it is impractical to demonstrate individual effects through RCTs, but such interventions may be cost-effective even without evidence demonstrating efficacy [39].

It is also important to consider the points of view of public health professionals, which we did not include in this study. However, a useful qualitative study by van der Graaf which explored how public health professionals view and engage with research, identified three main barriers when trying to engage with researchers: 1) differences in timescales; 2) limited budgets; and 3) difficulties in identifying appropriate researchers [40]. Some of the challenges associated with these barriers could be overcome with better communication and relationships between researchers and practitioners. In addition to providing the appropriate form of evidence, stakeholder engagement also plays a major part of facilitating impact as observed in our case studies. Researchers we interviewed we were able to ascertain the importance of high quality engagement with stakeholders such as local authorities and public health practitioners. They also acknowledged that given the interdisciplinary nature of complex public health problems, more inter-sectoral collaboration is needed. This may imply continuous shifts in the way financial resources are allocated at the local authority level to support public health activity, and working with initiatives in transport, social care, and education to tackle public health issues.

### Lessons for future research impact analyses

Supplementing our analysis of NIHR projects with these in-depth case studies revealed the extent to which impact pathways can diverge from the more linear models of impact that have informed understandings of impact processes in the past. Although population health improvement may be considered the endpoint for public health research impact, short and medium-term changes that facilitate this goal can also be considered impacts arising from this field [9]. The question of how these short and medium-term impacts should be accounted for in research impact analyses is a prominent feature of the literature on this subject [25, 41]. Further, while participants tended to focus on more direct, quantifiable forms of impact such as incorporation into policies or guidelines, our case studies also drew out several examples where participants had achieved less tangible forms of impact. This included impacts achieved through informal networks, non-academic publications and those coproduced with research participants. This also carries implications for how impact is understood and measured, especially in cases where research has a qualitative component which often produces less direct pathways to impact. As Greenhalgh and Fahy [6] have noted, the emphasis of the REF impact case study format on measurable impacts are such that these examples of research involving more complex and indirect pathways to impact are rare. Our analysis suggests that improving the recording and understanding of less tangible pathways to impact would render these more visible, and increase the likelihood of public health research being translated into practice in ways that benefit patients and the public.

## Conclusions

In addition to the School of Public Health and PHR, the NIHR supports public health research through a variety of other mechanisms, especially the HTA and HS&DR funding streams. Impacts from public health research were observed in a wide range of disciplinary areas. Our in-depth case studies highlighted a range of impact mechanisms including relationships with external stakeholders, targeted dissemination methods, getting the timing and type of evidence right and perseverance when disseminating negative findings. Interviews with PIs and other researchers associated with the projects also pointed to the pivotal role played by researchers’ own assumptions and initiatives in determining research impact. To support impacts from public health research, we encourage the acknowledgement and measuring of impact at different stages of the impact pathway, including localised impacts, and the difference in types of evidence required for community and local authority-based impacts. Finally, we note the importance of building capacity and resources to support impacts from public health research, and would like to see greater intersectoral cooperation in order to ensure that public health research fulfils its potential to effect positive changes at both national and local levels.

## Supplementary information

**Additional file 1.** Topic guide for interviews.

## Data Availability

Data was provided by the National Institute of Health Research (NIHR) for the purposes of this research. Requests to view will be referred to NIHR.

## Abbreviations

DH: Department of Health
EME: Efficacy and Mechanisms Evaluation
HS&DR: Health Services and Delivery Research
HTA: Health technology Assessments
I4I: Invention for Innovation
NIHR: National Institute of Health Research
PGfAR: Programme Grants for Applied Research
PHO: Public Health Overview
PHR: Public Health Research
PI: Principal Investigator
REF: Research Excellence Framework
RfPB: Research for Patient Benefit
SRP: Systematic Reviews Programme
TCC: Trainees Coordinating Centre
WHO: World Health Organisation

## Footnotes

1 https://www.ukri.org/innovation/excellence-with-impact/

2 http://impact.ref.ac.uk/CaseStudies/

3 Researchfish is an online platform that enables research funding organisations to capture and track the impact of their investments, and researchers to log the outputs and outcomes of their work. Available at: https://www.researchfish.net/home
