# Supplementary material for: Mechanisms and pathways to impact in public health research: a preliminary analysis of research funded by the National Institute for Health Research (NIHR)
Source: BMC Med Res Methodol. 2020 Feb 19;20:34. doi: 10.1186/s12874-020-0905-7 (PMC7031933; doi:10.1186/s12874-020-0905-7)
Supplement: Supplementary file 1 — Additional file 1. Topic guide for interviews. [file 12874_2020_905_MOESM1_ESM.docx]

**Additional file 1**

**Annex A**

Interview topic guide: Mapping the Non-Academic Impact of Public Health

1. Could you tell us a bit about your project <insert project> and your role with the project?

- How did you become involved?
- What was the rationale behind the project?
- How does this piece of research fit into your wider portfolio/research interests?

2. Did you have any collaborators on the project?

- Academic
- Outside of academia, for example charities, clinicians, hospital trusts, industry?

3. What did your project find?

4. How did you share these findings?

5. Thinking beyond academia, what in your view was the benefit or impact of the activity EG

- Training (of researchers, practitioners, others)
- Policy influences?
- Did the research influence a guideline? If so at what level?
- Overall, Health impacts?
- Societal change (change in perception/understandings of health-related behaviour/ change in practice)
- Changes in industry?
- Anything else?

6. How do you know the project had the impact you describe? What evidence is there? Eg Policy documents, Reports, Websites, Commentary or feedback from stakeholders

7. For each category discussed above, where impacts have been achieved from the research, who are the main beneficiaries?

- Which groups of people have benefitted eg. Patients, clinician groups (Eg cardiovascular surgeons etc)
- How widespread was the impact? Eg did it affect large groups/multiple groups? (geography and estimate of volume)

8. Which stakeholders were involved in facilitating the impact for this work?

- For example was the media involved, or policymakers, etc?
- How did they facilitate the impact?

9. What do you hope will happen going forwards?

10. What do you think was the most effective way in which impact was achieved? And did you encounter any problems or barriers to pursuing the intended impact?
